# Long-term complete response to intrathecal trastuzumab in a patient with leptomeningeal carcinomatosis due to her2- overexpressing breast cancer: Case report

**DOI:** 10.1097/MD.0000000000018298

**Published:** 2020-01-03

**Authors:** Francisco José Valdivia García, Natalia Palazón Carrión, Luis de la Cruz-Merino

**Affiliations:** Clinical Oncology Department, Medicine Department, Hospital Universitario Virgen Macarena, University of Seville, Seville, Spain.

**Keywords:** breast, cancer, HER2, intrathecal, leptomeningeal carcinomatosis, positive, trastuzumab

## Abstract

**Introduction:**

Leptomeningeal dissemination due to HER2-overexpressing breast cancer is a rare and hard to treat complication with short-term dismal prognosis.

**Patient concerns:**

A 34-year-old female previously treated because of HER2+ breast cancer is admitted to the Neurology Department in December 2016 due to sensory-motor neurological semiology.

**Diagnosis:**

A wide set of diagnostic tests is performed and finally cytologic findings after repeated CSF confirm leptomeningeal infiltration by breast carcinoma (panCK+, GATA3+).

**Interventions:**

Weekly intrathecal triple therapy with methotrexate, cytarabine and hydrocortisone plus trastuzumab is carried out during 4 months.

**Outcomes:**

Clinical and pathological response that lasts more than 24 months.

**Conclusion:**

Leptomeningeal carcinomatosis is an oncological situation where conventional therapies have limited activity. In HER2+ advanced breast cancer patients, intrathecal therapy with anti-HER2 therapy (trastuzumab) is feasible and may reach long-term disease control, especially in cases of low-tumor burden.

## 1 Introduction

### 1.1 Leptomeningeal carcinomatosis (in context)

Leptomeningeal carcinomatosis is one of the most feared complications in oncology as it carries almost universally a very dismal prognosis, with poor overall survival and few therapeutic options available. In breast cancer (BC), leptomeningeal carcinomatosis is associated with median overall survival that ranges from 2.2 to 4.4 months, being HER2+ the subtype with a slightly better prognosis.^[1]^ Anti-HER2 therapies have revolutionized the clinical scenario of HER2+ BC, being the monoclonal antibody targeted against the HER2 receptor trastuzumab, the backbone of any therapeutic strategy considered for this subgroup of patients.^[2]^ However, in HER2+ BC patients, one of the weak spots continues to be the increasing risk in developing intracranial disease (6.8% risk in 10 years), which includes leptomeningeal disease, a condition that might happen in 15% of these cases, most likely due to the low penetration capacity of trastuzumab through the blood-brain barrier.^[3,4]^ Therefore, it is interesting to ascertain if there are other more effective approaches, like intrathecal therapy, to reach higher trastuzumab levels in the CSF (cerebrospinal fluid) that could eventually result in a better control of the disease. At this point, the scientific evidence is scarce, and it is mainly based in retrospective studies and case series (Table 1). Two clinical trials (phase I/II) are currently being carried out with pending results (NCT01373710; NCT01325207).

**Table 1:**
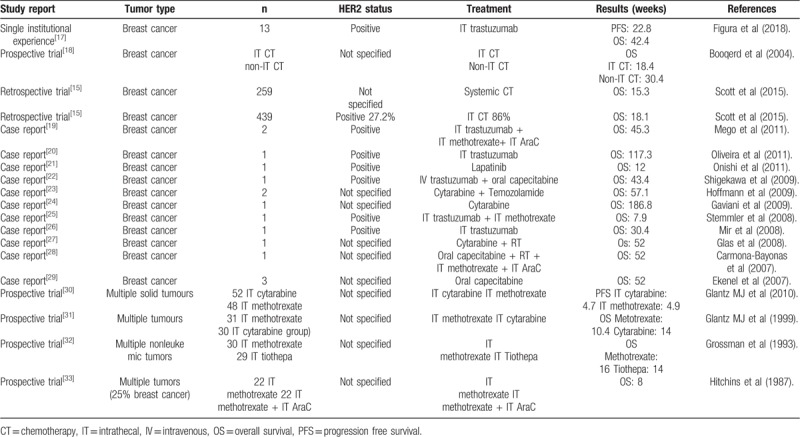
Synthesis of data in leptomeningeal carcinomatosis.

## 2 Patient consent

The patient has been informed and has given her consent for the publication of this case report.

## 3 Case report

We introduce the case of a 34-year-old woman, with no relevant family or personal history, that was diagnosed in September 2010, during the first quarter of her second pregnancy, with a 7 cm invasive ductal carcinoma with lobular pattern in the left breast. Immunohistochemical analysis showed a luminal-B HER2+ breast cancer (BC) subtype (ER 87%, PR 69%, c-erbb2 +++, Ki67- 62%). After diagnosis, pregnancy was interrupted and the patient received neoadjuvant chemotherapy as per the following: epirubicin + cyclophosphamide × 4 cycles (from October 2010 to December 2010) and docetaxel + trastuzumab × 4 cycles (from January 2011 to March 2011). After completion of neoadjuvant biochemotherapy a left radical mastectomy and an axillary lymphadenectomy were performed in April 2011. A pathological complete response was achieved in the breast whilst 2 lymph nodes out of 10 remained affected by BC metastases. Adjuvant strategy was completed with radiotherapy and hormone therapy with Tamoxifen (20 mg/day) during five years, combined with LHRH analogs for the first 2 years.

In December 2016 she is admitted in the Neurology Department because of dorsal and back pain, paresthesia and weakness in lower limbs ongoing for 2 weeks, with loss of sphincter control in the previous 48 hours. Physical examination revealed parapesis (3/5 in the left lower limb and 4/5 in the right lower limb) with kneecap and Achilles tendon areflexia. Once she is admitted, a wide set of complementary tests were carried out including complete blood count and biochemical analysis, urine analysis, cranial and vertebral column NMR, lumbar puncture with extraction of CSF, electromyogram of lower limbs, electroencephalogram, full-body computational tomography (CT) scan and analysis of antineuronal antibodies, all of them with negative results.

**Figure 1:**
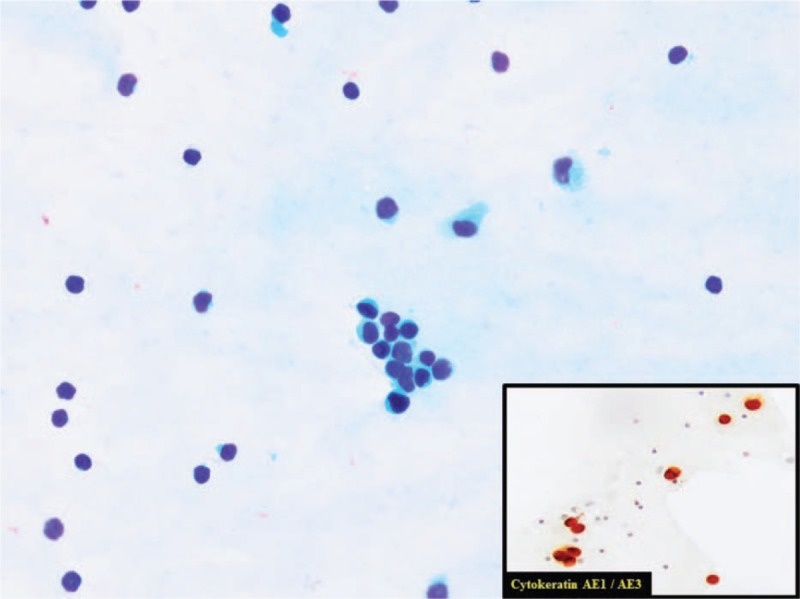
Cerebrospinal fluid with an infiltration by ductal breast carcinoma. Isolated cells and poorly cohesive cluster of cells. Eccentric nuclei often protruding from the cytoplasm. Enlarged, variably hyperchromatic nuclei in a clean background. In the image in the lower right corner, we can see positive immunoreaction for Cytokeratin AE1/AE3. This is concordant with an infiltration by carcinoma. Alvaro Gutierrez Domingo, MD, Pathological Department, Virgen Macarena Hospital, Sevilla (Spain).

**Figure 2:**
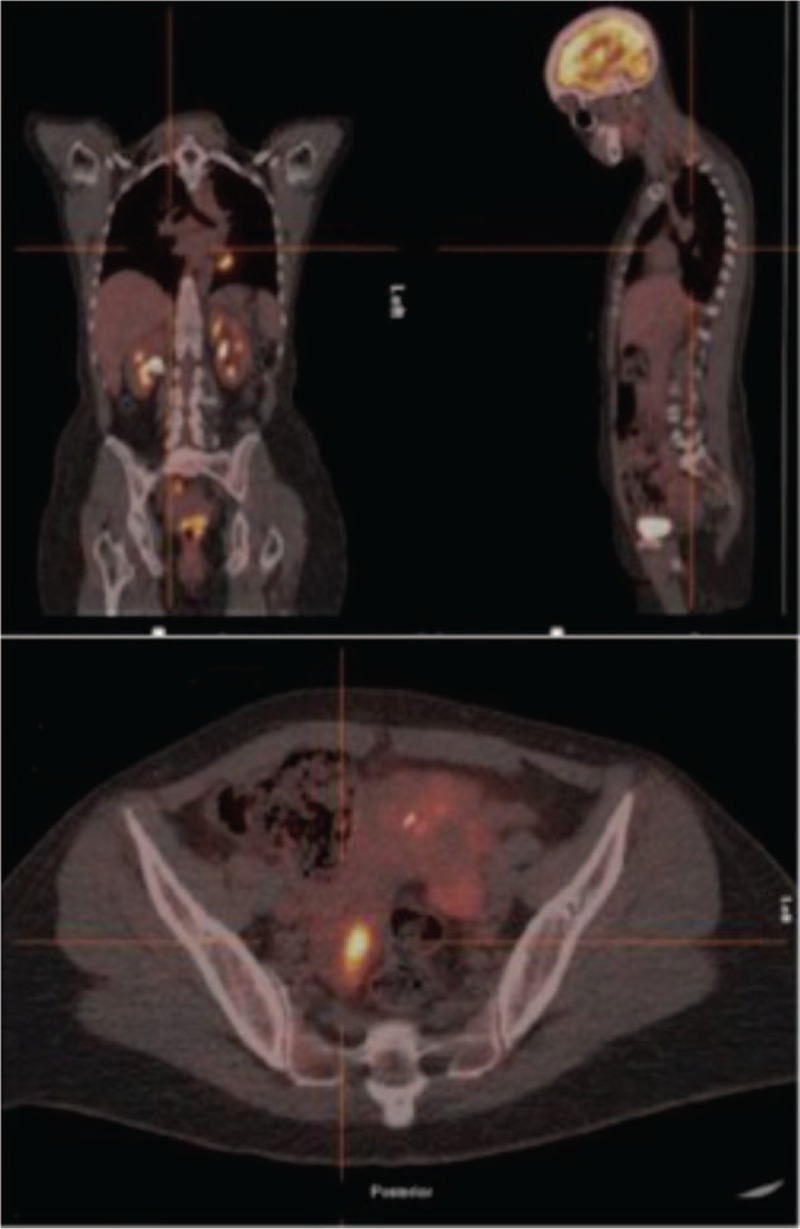
PET-CT with uptake in right hemipelvis.

On January 27^th^, 2017 a second extraction of CSF is performed and this time cytology confirmed infiltration by breast carcinoma (panCK+, GATA3+) (Fig. 1). Diagnosis of leptomeningeal carcinomatosis is assumed and the patient is transferred to the Oncology Department where a PET-CT is performed. The PET-CT showed an uptake in the right hemipelvis which is interpreted as physiological uptake in the ovary (Fig. 2).

Initially no extrameningeal disease is considered and therefore, at least with a palliative intent, weekly intrathecal biochemotherapy is initiated as per the following: a first vial with 40 mg of trastuzumab in 5 ml and a second vial with hydrocortisone 20 mg + cytarabine 30 mg + methotrexate 12 mg in 5 ml. After the second infusion, the patient was discharged on March 1st 2017 due to positive clinical evolution. Weekly intrathecal triple therapy with trastuzumab and cytology of CSF to monitor response were carried out in the following weeks. In March 2017 and, assuming clinically HER2+ disease that could not be confirmed by cytology, the patient started treatment with lapatinib 1250 mg every 24 hours. Afterwards, with the aim of ovarian castration, a bilateral salpingo-oophorectomy was performed in April 2017. The histopathology results revealed breast carcinoma infiltration in the right ovary with the following immunohistochemical results: 100% ER+, 100% PR+, HER2+++, 20% Ki67. In April 2017 letrozole 2.5 mg every 24 hours is used together with lapatinib.

**Figure 3:**
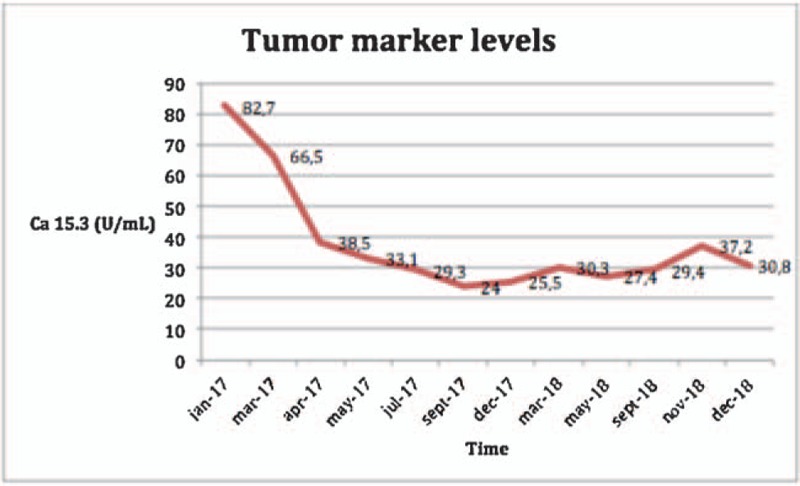
Evolution of tumor marker levels.

Progressively the patient recovered autonomy and the dorsodynia disappeared with a better functional performance. The tumor markers decreased (Ca 15.3 82.7 UI/ml in January 2017; UI/ml in April 2017; normal range <25–40 UI/ml) (Fig. 3).

**Figure 4:**
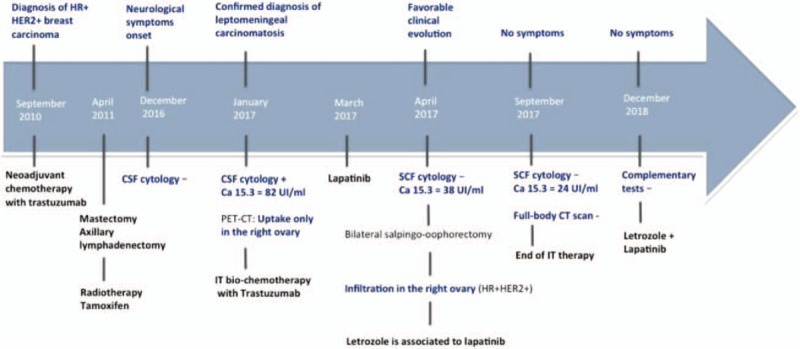
Clinical evolution of the patient.

For the first time on April 20^th^, 2017 (since January) cytology in SCF was negative, which is confirmed in subsequent SCF punctions. From May 2017 intracranial hypotension is detected and so the intrathecal therapy continues but in a three-week manner until September 2017. From there on the therapy is no longer feasible due to the inability to extract SCF and the patient showed no symptoms, following oral treatment with letrozole + lapatinib, having excellent functional abilities and being considered free of disease after 31 months from the initial diagnosis of leptomeningeal carcinomatosis (Fig. 4).

## 4 Discussion

The appearance of metastasis in the central nervous system is an event with a higher frequency in patients with HER2-overexpressing breast cancer with respect to other BC subtypes (30% vs 10%) because of increased affinity to the CSN by HER2-overexpressing breast cancer.^[5,6]^ In 15% of the cases, this condition appears because of leptomeningeal invasion, which makes the prognosis even worse.^[7]^ Furthermore, because of its low frequency, especially in the absence of brain parenchymal metastasis, the therapeutic approach is limited and the prognosis is devastating, with a median survival of 4.5 weeks,^[8]^ so it is indeed considered a real clinical challenge.

Therapies based on trastuzumab, a monoclonal antibody targeted against the HER2 receptor, are considered the best option in treatment against HER2-overexpressing breast cancer. The data about its ability to break through the brain-blood barrier are scarce and contradictory,^[9,10]^ but in any case the levels reached in SCF are slightly lower than the concentration in blood, even in patients treated with radiotherapy with known disruptive effect of the brain-blood barrier.^[11]^ Thus, intrathecal therapy seems attractive specially in cases like our patient's, in which there was no clear evidence of disease at any other level, or at least a low tumor burden. No consensus exists on the adequate dosing and frequency of IT trastuzumab but dose ranges from 5 to 150 mg twice a week, weekly or every 3 weeks are the most used schedules in scientific literature.^[12,13]^ IT trastuzumab alone or as part of combination therapies seem to be safe without serious adverse events. Clinical improvement and CSF response seem to be associated with longer PFS because of stabilization of the CSN disease.^[14]^

The more comprehensive systematic review published up to now to evaluate the efficacy of intrathecal treatments in leptomeningeal carcinomatosis include the analysis of 36 studies (case reports, prospective trials and retrospective studies) with a total of 851 patients with BC.^[15]^

From all the breast cancer cases included in this review,^[15]^ only 15% of them show results from prospective trials. However, none of these prospective trials states the HER2 status and even though intrathecal therapy was carried out in 86% of the cases, none of them used anti- HER2 intrathecal therapy. A median overall survival of 14.9 weeks is reported. Likewise, in the group of retrospective studies,^[15]^ intrathecal treatment was performed in 86% of the cases but in none of them trastuzumab was used (59% methotrexate, 29% liposomal cytarabine). Clinical response rates were observed in 68% and an overall median survival in BC cases of 18 weeks. Furthermore, those factors that could potentially impact the survival of patients with leptomeningeal breast cancer were analyzed. Thus, the control of oncologic disease or the lack of metastases outside the CNS, the use of intrathecal treatment, the use of intravenous systemic treatment and the clinical response seem to be factors that contribute favorably to overall survival. As unfavorable factors, ECOG>2, histologic grade 2 to 3 and negative or triple negative breast cancer^[15]^ were considered.

A group of case reports reflects that intrathecal chemotherapy with trastuzumab, methotrexate and/or cytarabine as single agent or combined is feasible and may reach a high rate of response^[10,15,16]^ with prolongation of disease control and probably survival in HER2+ patients.

An American single-center retrospective study^[17]^ recently published included 13 patients with HER2+ breast leptomeningeal disease and parenquimal brain metastases treated with intrathecal trastuzumab between November 2012 and May 2017 until November 2017. The average age was 48 and most of them (92%) had received previous cranial radiotherapy. The administered dose of trastuzumab was 20 to 50 mg twice a week for 4 weeks, then once a week for 4 weeks and finally a monthly maintenance dose of 80 mg. The average time observed until leptomeningeal progression was 5.7 months. Four patients reached a progression-free survival above 6 months and one of them had a response that lasted more than 5 years. Regarding tolerance, this approach was considered feasible with no significant adverse effects.^[17]^

At the moment, there are 2 ongoing phase I/II clinical trials which try to evaluate the efficacy and safety of the intrathecal use of trastuzumab in patients with leptomeningeal carcinomatosis due to HER2-overexpressing breast cancer (NCT01373710; NCT01325207).

As it can be noticed, the scientific evidence about intrathecal treatment with trastuzumab is very scarce and it is based on case series and some retrospective studies with their associated biases and limitations. Despite that, intrathecal therapy with trastuzumab should be considered in this setting, due to the safety and potential benefit observed in some cases, with an overall survival reported that is certainly higher than what it would be expected for the available data. Furthermore, it is important to highlight that in most of the cases (70%–89%), diagnosis of leptomeningeal disease coincides with a situation of active systemic disease or with patients with extensive brain damage, which was not the case of our patient. In the case reported, the low-tumor burden on one side, and the “intensive” therapeutic approach, including intrathecal trastuzumab combined with systemic therapy (Lapatinib plus Letrozole), then again, may explain the exceptional positive evolution with a progression-free survival that, up to now, lasts more than 20 months.

## Author contributions

**Conceptualization:** Francisco José Valdivia García, Natalia Palazón Carrión.

**Supervision:** Luis de la Cruz-Merino.

**Validation:** Luis de la Cruz-Merino.

**Writing – original draft:** Francisco José Valdivia García.

**Writing – review & editing:** Francisco José Valdivia García, Natalia Palazón Carrión.
